# Preserving Food

**Published:** 1875-05

**Authors:** 


### PRESERVING FOOD.

The season of furnaces is over, and now the season of refrigerators begins. We have been seeking for warm air and pure air all winter, and now we are looking for cool air and pure air, as well for our provisions as for ourselves. Lesley's Gothic Furnace seemed to us a most desirable thing as a warming device, and to-day we have looked at his Zero Refrigerator, and are satisfied to commend it to such of the readers of our Journal as are desirous of securing a proper cooling and preserving closet.

What is needed in a refrigerating box is *uniformity of temperature.* This is the all-important condition. There must be no fluctuation, no variableness. With the temperature at the zero of our clumsily-constructed thermometer, decay of meat is quite impossible, as is also fermentation. In the frozen zone of Siberia there have been found bodies of animals so situated, as to render it probable that they had been dead for generations. Their flesh was sound and sweet. The secret of their preservation was that no expansion of fibre had been suffered, by a varying temperature, with its power to rupture the tissues and commingle the juices which tend to fermentation and decay.

The same conditions are sought and reached by the Zero Refrigerator. It seems to matter not *how little ice* is placed in it, provided that it is never wholly without ice, during the hot season. The walls of the safe are filled with cork — a sweet, cleanly non-absorbent, non-conducting substance, as all can testify. Within this house of cork, all kinds of food are found to "keep," regardless of the condition of the mercury without. The pavement may be so hot as to blister our soles ; but we rest secure in the assurance that we shall find our milk, our fish, our vegetables, our butter and our strawberries, sweet, cool and delicious when we summon them from our Zero receptacle.

There are grand points about the construction of these Refrigerators. They are made of clear, well-seasoned lumber, and are filled with cork, as we have said. They are lined with zinc, have galvanized iron ice-box, good locks, with white knobs and assorted keys. They are supplied with tinned wire shelves, silver-plated faucet and castors, and are grained in oak.

The ice is placed in the centre of the Refrigerator through the top door, on a wooden rack several inches above the faucet. No communication is allowed between the ice and provision chamber, and no hot air is allowed to touch the ice, except as the door is opened for putting in more ice ; it being thus protected, will last longer than in the ventilating refrigerators, which are great ice consumers, and which introduce warm, moist air into the provision chamber. The drippings of the ice are retained and drawn off from the faucet. Housekeepers will be pleased with the arrangement, as there is no water running over the floor. When the ice is kept clean, and the wooden ice-rack boiled so as to remove the woody taste, the ice-water can be used as in the ordinary water cooler. Persons using this refrigerator will find that the consumption of ice is reduced to a minimum—a matter worthy of consideration. There are several other good points about the "Zero" to which we may hereafter refer. Just now we will only add that Mr. A. M. Lesley makes these Refrigerators by the thousand at his factory, No. 226 West 23d street, and that a little pamphlet which he mails to inquirers, will prove of interest to all who will read it.

